# Identification of Novel and Safe Fungicidal Molecules against *Fusarium oxysporum* from Plant Essential Oils: In Vitro and Computational Approaches

**DOI:** 10.1155/2022/5347224

**Published:** 2022-07-26

**Authors:** Qudsia Yousafi, Shabana Bibi, Shahzad Saleem, Abrar Hussain, Mohammad Mehedi Hasan, Maria Tufail, Amina Qandeel, Muhammad Saad Khan, Sania Mazhar, Maha Yousaf, Mahmoud Moustafa, Mohammed Al-Shehri, Mohammad Khalid, Atul Kabra

**Affiliations:** ^1^COMSATS University Islamabad, Sahiwal Campus, Sahiwal, Pakistan; ^2^Department of Biosciences, Shifa Tameer-e-Millat University, Islamabad, Pakistan; ^3^Yunnan Herbal Laboratory, College of Ecology and Environmental Sciences, Yunnan University, Kunming, 650091 Yunnan, China; ^4^Department of Biochemistry and Molecular Biology, Faculty of Life Science, Mawlana Bhashani Science and Technology University, Tangail, Bangladesh; ^5^PCSIR Laboratory Complexes, Lahore, Pakistan; ^6^Department of Biosciences, COMSATS University Islamabad, Islamabad 45550, Pakistan; ^7^Department of Biology, College of Science, King Khalid University, 9004 Abha, Saudi Arabia; ^8^Department of Botany and Microbiology, Faculty of Science, South Valley University, Qena, Egypt; ^9^Department of Pharmacognosy, College of Pharmacy, Prince Sattam Bin Abdulaziz University, Al-Kharj 11942, Saudi Arabia; ^10^University Institute of Pharma Sciences, Chandigarh University, Ghruan-140413, Mohali, Punjab, India

## Abstract

Phytopathogenic fungi are serious threats in the agriculture sector especially in fruit and vegetable production. The use of plant essential oil as antifungal agents has been in practice from many years. Plant essential oils (PEOs) of *Cuminum cyminum*, *Trachyspermum ammi*, *Azadirachta indica*, *Syzygium aromaticum*, *Moringa oleifera*, *Mentha spicata*, *Eucalyptus grandis*, *Allium sativum*, and *Citrus sinensis* were tested against *Fusarium oxysporum*. Three phase trials consist of lab testing (MIC and MFC), field testing (seed treatment and foliar spray), and computer-aided fungicide design (CAFD). Two concentrations (25 and 50 *μ*l/ml) have been used to asses MIC while MFC was assessed at four concentrations (25, 50, 75, and 100 *μ*l/ml). *C. sinensis* showed the largest inhibition zone (47.5 and 46.3 m^2^) for both concentrations. The lowest disease incidence and disease severity were recorded in treatments with *C. sinensis* PEO*. Citrus sinensis* that qualified in laboratory and field trials was selected for CAFD. The chemical compounds of *C. sinensis* PEO were docked with polyketide synthase beta-ketoacyl synthase domain of *F. oxysporum* by AutoDock Vina. The best docked complex was formed by nootkatone with -6.0 kcal/mol binding affinity. Pharmacophore of the top seven *C. sinensis* PEO compounds was used for merged pharmacophore generation. The best pharmacophore model with 0.8492 score was screened against the CMNP database. Top hit compounds from screening were selected and docked with polyketide synthase beta-ketoacyl synthase domain. Four compounds with the highest binding affinity and hydrogen bonding were selected for confirmation of lead molecule by doing MD simulation. The polyketide synthase-CMNPD24498 showed the highest stability throughout 80 ns run of MD simulation. CMNPD24498 (FW054-1) from *Verrucosispora* was selected as the lead compound against *F. oxysporum*.

## 1. Introduction

The secondary metabolites produced by plants play an important role in plant defense mechanism [1]. Most of the plant essential oils (PEOs) are secondary metabolites and found to be involved in plant defense system as antioxidant, antifungal, or antimicrobial [2]. Very large contribution of the PEOs has been reported in traditional medicine manufacturing for the last many decades [3]. PEOs have been commercially used in pharmaceutical, cosmetics, and beverage industry [4].

The need for use of biological agents in pest management has evolved due to irreversible and drastic effects of synthetic pesticides in the environment and human health [4]. The conventional biological pest control agents are parasitoid, predators, microbe, and fungi, but PEOs have also been assigned in this category because of their origin as plant secondary metabolites. They have become popular as an integral part of integrated pest management (IPM) because of the antimicrobial, antioxidant, and antifungal properties [5]. The antimicrobial properties of PEOs are rendered by their terpenoid and phenol constituents [6]. These compounds have been found safe for humans, animals, and environment, when used in food medicines and pesticides [7]. The use of PEOs, as a substitute of synthetic chemicals, has been recommended in the European Union directive vide 2009/128/CE [8].

More than 30% of the crops, from sowing till harvesting even in stored conditions, are caused by phytopathogenic fungi [9]. Various chemical products have been in use for controlling fungal plant disease. But the use of PEOs as an antifungal agent is getting popular in crop protection sector from the last two decades [10]. The *in vitro* assay of PEO can be performed by using various parameters. The efficacy of PEOs against phytopathogenic fungi can be tested in laboratory by evaluating, as lethal concentration (LC50), minimum inhibitory concentration (MIC), and minimum fungicidal concentration (MFC) [11].

The genus Citrus of Rutaceae family includes about seventeen species distributed throughout the tropical and temperate regions [12]. Its fruit is used as deserts, and the unique aroma of this plant is due to its essential oils present in the leaf, peel, and bark [13]. Various investigations reveal the antimicrobial, antifungal, antioxidant, and radical-scavenging properties of the biological active molecules found in these PEOs [14]. The essential oils from *Citrus limon* and *Citrus aurantifolia* have been reported as strong inhibiter of phytopathogenic fungi growth in different crops [15].

Tomato (*Lycopersicon esculentum*), belonging to the family Solanaceae, is an important crop grown worldwide. It is an important crop of summer and usually regarded as the pole of kitchen gardening, used in sauces and different food stuff compositions [16]. Tomato cultivation is affected by various kinds of pathogenic disease caused by viruses, fungi, bacteria, and mycoplasma. Various kinds of fungal diseases are responsible for affecting the tomato production worldwide which are early blight of tomato, late blight of tomato, and tomato wilt disease. According to an estimate, late blight and early blight of tomato are responsible for almost 49-91% yield losses in Pakistan [17].

Fusarium wilt is a soil-borne fungal disease of solanaceous plant caused by *Fusarium oxysporum* fungus. Fusarium wilt is very common in tropical southern areas during warm-to-hot weather [18]. Pathogen blocks the xylem vessels due to which plant wilts and dies off. Fungal pathogens can cause 80% of plant diseases [19]. Since antiquity, *F. oxysporum* f. sp. lycopersici, as with all phytopathogenic fungus, has posed a hazard to agriculture [20]. Chemical fungicides, while commonly employed, are costly and polluting and provide a danger of toxicity to the planters and hence do not form a crop management technique for long-term development [21].

The use of computational approaches, of computer-aided drug design (CADD), is getting popular in pesticide development research and industry from the end of the last decade [22]. The increasing rate of pesticide resistance development, human health hazards, and environment pollution by synthetic pesticides tends to an urge of development of novel effective and safe molecules for agricultural industry. The pesticide development in the wet labs is a tedious expensive and time-consuming job. The pipeline of techniques used in CADD is based on the initial screening of chemical compounds which leads to narrowing down the dataset consisting of the effective and potential molecules [22]. This regime changes in pesticide development from conventional methods to combining with computational technology have been proved a progressive trend [23]. These fast and smart strategies of computer-aided pesticide design (CAPD) can be useful in lead molecule identification. To avert the emergence of pesticide resistance, CADD techniques deliver baseline knowledge regarding potentially safer pesticide compounds and their target site [8].

The currently planned study was targeted to detect *in vitro* antimicrobial effectiveness of five medicinal PEOs against *Fusarium oxysporum*. In the first step of the study, MIC and minimum fungicidal concentrations were evaluated for ten essential oils against *F. oxysporum*. Secondly, three PEOs showing good results in the first step were tested as foliar application and seed treatment; lastly, one of the qualified essential oils was used for computer-aided fungicide design. In the current study, we not only have identified a new, safe, and effective antifungal molecule against *F. oxysporum* but also new target site. This can become helpful in pesticide resistance management due to target site insensitivity. These findings are recommended to be confirmed in wet lab experiment and can be used in novel, safer, and effective fungicide against *F. oxysporum*.

## 2. Materials and Methods

The seeds (*Allium sativum*, *Trachyspermum ammi*, *Cuminum cyminum*, and *Syzygium aromaticum*), peel (*Citrus sinensis*), and leaves (*Azadirachta indica*, *Moringa oleifera*, *Mentha spicata*, and *Eucalyptus grandis*) of nine plants were collected locally for PEO extraction. The PEO extraction was done by following methods described. The extracted PEOs, following methods recommended by Odak et al. [24], were stored at room temperature. The fungal culture of *F. oxysporum* used in the experiment was provided by the PCSIR, Lahore (Pakistan). The fungus was subcultured at room temperature (25°C) for 120 hours using potato dextrose agar (PDA) slants to prepare spore suspension for subsequent experimental use.

### 2.1. Laboratory Evaluation of Plant Essential Oil Activity

#### 2.1.1. Minimum Inhibitory Concentration (MIC) and Minimum Fungicidal Concentration (MFC) Evaluation of Plant Essential Oils

The agar well diffusion method was used to evaluate MIC for two concentrations (25 and 50 *μ*l/ml) of PEOs. The size of inhibition zone produced by PEO application reflects the inhibitory concentration of the PEO. The lowest concentration of essential oil producing the largest inhibition zone reflects it potential to inhibit fungal growth, and this concentration is referred as the MIC. Each treatment was replicated three times in CRD experiment layout.

The test tubes containing culture media broth and essential oil (at three concentrations 50, 75, and 100 *μ*l/ml) to be tested were inoculated with 1 × 10^6^ cfu/ml fungal spore load. Broth tubes used without essential oils were assigned control. The fungal growth was observed in test tubes 48 hours after incubation at 25°C. A volume of 100 *μ*l, from tubes showing no visible fungal growth, along with agar was poured in petri plates after the incubation duration. The lowest PEO concentration showing no fungal growth in petri plates, after 48 hours of incubation, was considered as MFC.

### 2.2. Field Trials

#### 2.2.1. Foliar Application of Plant Essential Oils

Two concentrations, 60 and 80 *μ*l/ml, of *E. grandis*, *C. cyminum*, and *C. sinensis* PEO were selected to be applied as foliar spray in the field experiment. Treatments (Supplementary Table1) were planned under completely randomized design with three replications. A fungal spore load of 10^6^ cfu/ml was inoculated in soil as pathogen inoculum at 15 days posttransplant stage. The foliar applications of PEOs were done four times with 15 days interval.

*(1) Data Recording*.

1. Percent disease incidence: the plants showing symptoms of Fusarium wilt were counted to record percent disease incidence using following formula [25]

(1) $$\begin{array}{c} \text{Percent disease incidence}=\frac{\text{Number of infected plant }}{\text{Total number of plants assessed }}\times 100. \end{array}$$

(2) Percent disease severity: a scale of 0-5 was kept as standard to calculate the disease severity as suggested by Rahman et al. [26]. A standard formula by Chester [27] was followed for the determination of percent disease index (PDI)

(2) $$\begin{array}{c} \text{PDI}=\frac{\text{Sum of all numerical ratings}}{\text{Total number of plants }\left(\text{sample}\right)}\times \text{highest rating}\times 100. \end{array}$$

Percent disease severity (PDS) was calculated to assign the disease severity scales/ratings to the treatment. Ten infected leaves were randomly selected from ten infected plants each. Total leaf area was measured by using regression equation (*R*^2^, 98) developed by Blanco and Folegatti [28].

(3) $$\begin{array}{c} \text{Total leaf area}=0.347\cdot \left(L.W\right)-10.7. \end{array}$$

Percent disease severity (PDS) for each treatment was calculated as under, by observing and measuring the infected leaf area [29].

(4) $$\begin{array}{c} \text{PDS}=\frac{\text{Infected leaf area}}{\text{total leaf area}}\times 100. \end{array}$$

The following formula was used to calculate percent efficacy of disease control (PEDC) of each treatment [30].

(5) $$\begin{array}{c} \text{PEDC}=\frac{\text{Infection index in control}-\text{Infection index in treatment}}{\text{Infection index in control }}\times 100. \end{array}$$

#### 2.2.2. Tomato Seed Treatment

Twenty preinoculated seeds were treated with 1 ml of 60 *μ*l/ml concentration of PEOs (*C. sinensis, E. grandis*, and *C*. *cyminum*). Treatments with fungicide and sterile distilled water served as controls. The experiment was performed using Randomized Complete Block Design (RCBD) with five treatments and three replications (Supplementary Table 2).

Land preparation and agronomic field practices were done as directed by the Punjab Agriculture Department, Pakistan. A fungal spore load of 10^6^ cfu/ml was inoculated in soil as pathogen inoculum at 15 days posttransplant stage.

*(1) Data Recording and Analysis*. The data for PDI and PDS were recorded fortnightly as mentioned in foliar application experiment. Fruit was harvested and weighed from randomly selected ten plants for each treatment. Plant height was recorded from randomly selected 10 plants from each treatment. Fruit parameters, fruit weight, pericarp thickness, and fruit volume of randomly selected 20 fruits from each treatment were recorded. Fruit volume was measured by using the regression equation (*R*^2^, 98) developed by Concha-Meyer et al. [31] as

(6) $$\begin{array}{c} \text{Tomato fruit volume }\left({\text{cm}}^{3}\right)=7.3+0.92\text{ Weight }\left(\text{g}\right). \end{array}$$

Analysis of variance (ANOVA) was done to find significant difference among treatments. Multiple comparison among the treatments to find statistical differences or similarities among the treatments was done by using Tukey's HSD test.

### 2.3. Computer-Aided Fungicide Designing (CAFD)

Laboratory- and field test-qualified essential oil was selected for identification of potential lead molecule against *F. oxysporum.*

The chemical compounds of essential oil of orange *Citrus sinensis* L. were retrieved from literature, and their structure were retrieved from PubChem (Supplementary Table 3). The pesticide likeness [32] and nontoxicity of the compounds were predicted by DruLiTo 2.0 and DataWarior. The qualified compounds were selected for further use in docking.

#### 2.3.1. Protein 3D Structure Prediction and Molecular Docking

The amino acid sequence of polyketide synthase, an important toxin-producing enzyme in *F. oxysporum* [33], was retrieved from UniProt (UniProt ID: A0A0D2YG10). The toxin-producing domain of enzyme was predicted by InterPro-EMBL-EBI. Three-dimensional structure/model of the selected domain was predicted by using online server Robetta. The predicted 3D model was refined by an online available server GalaxyWEB, and the refined structure was evaluated by SAVES server.

The molecular docking of polyketide synthase domain and selected *C. sinensis* PEO compounds/ligands was done by using AutoDock Vina.

#### 2.3.2. Pharmacophore Modeling and Virtual Screening

The docked ligand-receptor complexes showing lower binding energies and hydrogen bond interaction were selected to be imported, as training set, to Ligand Scout 4.4 for pharmacophore generation. The best pharmacophore model was selected for virtual screening against Comprehensive Marine Natural Products Database (CMNPD). The matching compounds were evaluated and screened as in the first docking.

#### 2.3.3. Molecular Dynamics (MD) Simulations

Four protein-ligand docked complexes having lower binding energy were used for MD simulation. The software package, Amber v18, was used for MD simulations at 80-nanosecond time period.

## 3. Results

### 3.1. Laboratory Evaluation of Essential Oil Activity

#### 3.1.1. Minimum Inhibitory Concentration (MIC) and Minimum Fungicidal Concentration (MFC) Evaluation of Plant Essential Oils

**Figure 1:**
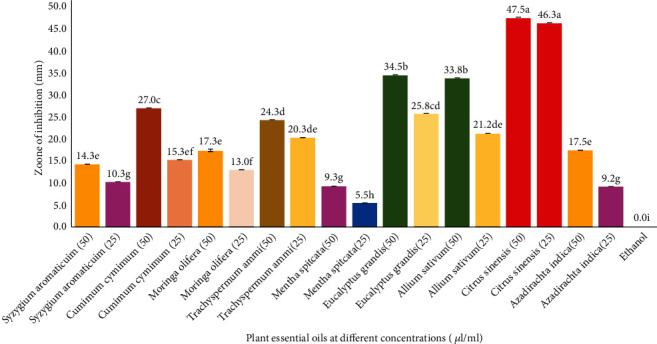
Minimum inhibitory concentration of different concentrations of plant essential oils against *Fusarium oxysporum*.

**Figure 2:**
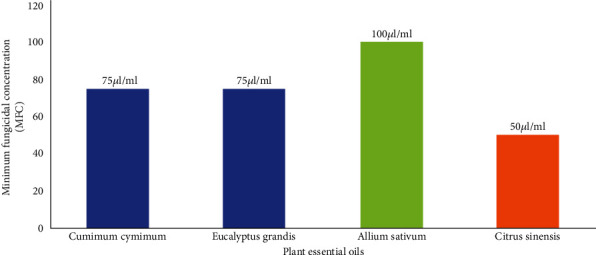
Minimum fungicidal concentration activity of different plant essential oils against *Fusarium oxysporum*.

The significantly largest but not significantly different from each other inhibition zone, 47.5 and 46.3 mm, was recorded for *C. sinensis* 50 *μ*l/ml and *C. sinensis* 25 *μ*l/ml, respectively (Figure 1). No fungal colony growth after 72 hours was observed in *A. sativum* 100 *μ*l/ml, *E. grandis* 75 *μ*l/ml, *C. cyminum* 75 *μ*l/ml, and *C. sinensis* 50 *μ*l/ml treatments (Figure 2).

### 3.2. Field Trials

#### 3.2.1. Foliar Application of Plant Essential Oils

**Figure 3:**
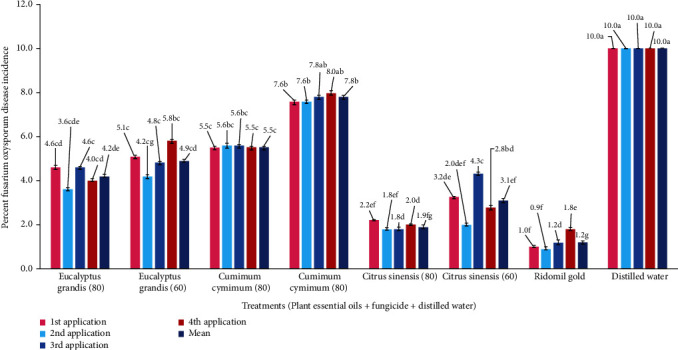
Effect of foliar application of different concentrations of plant essential oils on percent disease incidence of *Fusarium oxysporum* in tomato.

*(1) Percent Disease Incidence*. The percent disease incidence after the first application was significantly the lowest for the plant essential of *C. sinensis*; 80 *μ*l/ml showed significantly the lowest (2.2) percent disease incidence (Figure 3). In the mean percent disease incidence after four applications of essential oil, only *C. sinensis* 80 *μ*l/ml showed the significantly lowest (1.9) percent disease incidence. A not significantly different percent disease incidence was observed among the rest of the treatments. A not significantly different trend of disease incidence was observed among different application intervals.

*(2) Percent Disease Severity*. The data for percent disease severity were recorded two times after PEO application, i.e., after the first and fourth applications (Table 1). The significantly lowest percent disease severity (3.1), after the first PEO application, was recorded for *C. sinensis* (80 *μ*l/ml), which was not significantly different from those for *C. sinensis* 60 *μ*l/ml, *E. grandis* (80 *μ*l/ml), and Ridomil Gold. Two treatments (*C. sinensis* 80 *μ*l/ml and *C. sinensis* 60 *μ*l/ml) fell in class 1 of disease severity scale with PEDC value 80 and PDI 20. A similar trend for percent disease severity was observed after application.

#### 3.2.2. Tomato Seed Treatment

The efficacy of essential oil was tested as seed treatment. As the PEOs are volatile in nature, it might be possible that they act more effectively as seed treatment than foliar spray application [34].

*(1) Percent Disease Severity and Percent Disease Incidence*. The data were recorded from ten randomly selected plants (Table 2). All the three treatment effects on disease severity scale fell in class 1. The lowest (2.11) percent disease severity, not significantly different from that by *E. grandis*, was recorded for *C. sinensis*. Both treatments fell in DSS class 1, while percent disease incidence was significantly the lowest (1.5) for *C. sinensis*.

**Figure 4:**
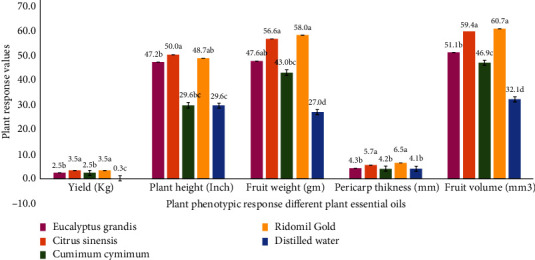
Tomato plant phenotypic response to the seed treatment by plant essential oils.

*(2) Effect of Seed Treatment on Yield, Plant Height, and Fruit Characteristics of Tomato*. The significantly highest fruit yield per plant (3.8 kg) was harvested from the plants treated with *C. sinensis* oil (Figure 4). The fruit yield per plant was not significantly different among the treatments *E. grandis* and *C. cyminum.* Plant height was significantly the highest for *C*. *sinensis*. The significantly highest value (59.4 m^3^) for fruit volume was recorded in plants treated with *C. sinensis* PEO.

### 3.3. Computer-Aided Fungicide Design (CAFD)

#### 3.3.1. Chemical Compound Structure Acquisition and Screening

**Figure 5:**
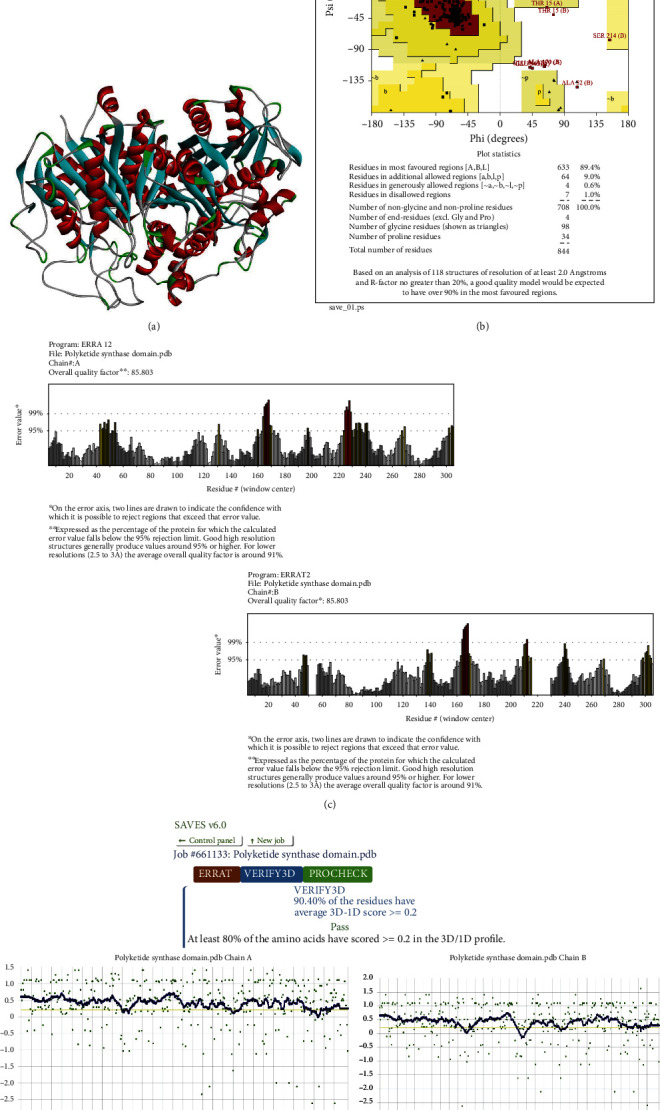
Refined 3D model of polyketide synthase beta-ketoacyl synthase domain in *Fusarium oxysporum* and model evaluation results: (a) refined 3D model, (b) Ramachandran plot, (c) ERRAT score, and (d) verify 3D score.

Only thirty-three compounds were qualified to have pesticide likeness and nonhazardous for humans (Supplementary Table 4). Active domain beta-ketoacyl synthase (IPR020841) of polyketide synthase was selected from domain scanning results. The refined 3D model passed the quality checks and was saved for further analysis (Figure 5(a)). The overall Ramachandran score of the refined 3D model of domain was 98.4% (Figure 5(b)). Among which, 89.4% amino acid residues were found in the most favored region while only 9% residues lied in the additional allowed region. ERRAT quality factor was 85.803 Figure 5(c). The residues passed the verification 3D check with 90.40% residues having averaged 3D-1D score ≥ 0.2Figure 5(d).

#### 3.3.2. Molecular Docking

Only sixteen compounds having hydrogen bond donor (HBD)/hydrogen bond acceptor (HBA) sites were selected to perform ligand-receptor docking. The overall binding affinity among docked complexes ranged from -6.2 to -4.3 kcal/mol (Table 3). The highest binding affinity (-6.2) resulted for two compounds: caryophyllene oxide and germacrene. But no hydrogen bond was found in both cases. Only seven ligand molecules showed hydrogen bonding with the protein. One hydrogen bond was formed in six docked complexes while nootkatone formed two hydrogen bonds with -6.0 kcal/mol binding affinity.

#### 3.3.3. Pharmacophore Modeling, Virtual Screening, and Molecular Docking

**Figure 6:**
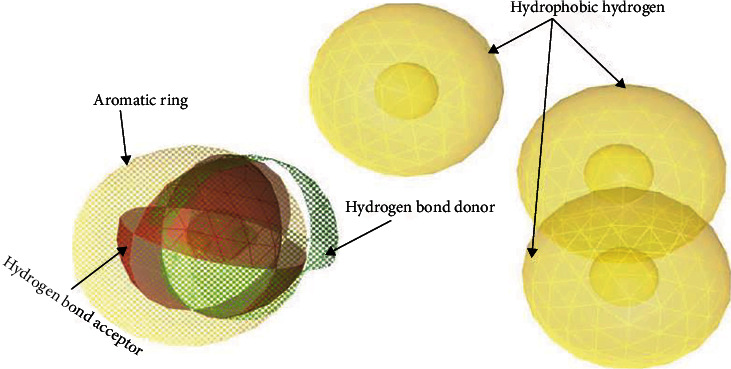
Pharmacophore features of selected, polyketide synthase beta-ketoacyl synthase domain in *Fusarium oxysporum* inhibitor, compounds from *Citrus sinensis* plant essential oils.

Seven chemical compounds showing low binding energies and hydrogen bond interaction, *α*-terpineol, (Z)-p-menth-2-en-1-ol, neral, elemol, nootkatone, and citronellyl acetate, were selected for merged feature pharmacophore generation. The best pharmacophore model (score: 0.8492) was selected for virtual screening. The selected pharmacophore had two HBA, two HBD, three hydrophobic hydrogens, and one aromatic ring (Figure 6). The hit rate 20.35% was obtained in virtual screening of the pharmacophore against CMNPD. Only 27 compounds, after screening, were qualified to be docked against query protein. The binding affinity of ligand-protein docked complex ranged from -6.9 to -4.1 (Table 4).

#### 3.3.4. Molecular Dynamics Simulation

**Figure 7:**
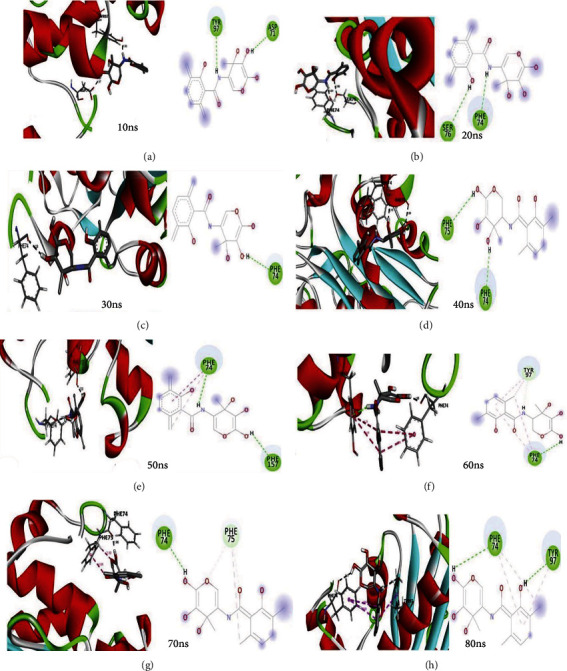
(a–h) Three-dimensional (3D) and two-dimensional (2D) interactions of polyketide synthase beta-ketoacyl synthase domain-CMNPD24498 at different time slots during MD simulation.

**Figure 8:**
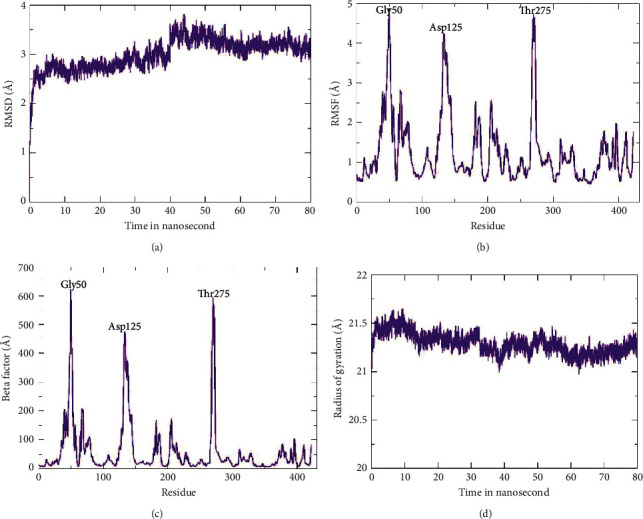
MD simulation results of polyketide synthase beta-ketoacyl synthase domain-CMNPD24498 complex.

Four protein-ligand docked complexes with lower binding energy, i.e., beta-ketoacyl synthase-CMNPD91 (-6.9 kcal/mol), beta-ketoacyl synthase-CMNPD19958 (-6.7 kcal/mol), beta-ketoacyl synthase-CMNPD1118 (-6.5 kcal/mol), and beta-ketoacyl synthase-CMNPD24498 (-6.2 kcal/mol), were simulated in an explicit water environment for a total of 80 ns. The beta-ketoacyl synthase-CMNPD24498 (-6.2 kcal/mol) complex showed stable interactions throughout the run. Three hydrogen bonds were found originally in the complex which was retained till 80 ns. At 30, 40, 60, and 70 ns, only one hydrogen bond was observed (Figures 7(a)–7(h)). Phenylalanine was found, most of the time, to be involved in hydrogen bond formation. To test the simulation system reliability, the backbone atom deviation was measured using the RMSD (root-mean-square deviation). In particular, the RMSD result revealed that the graph exhibited a progressive increase starting at 1 Å and oscillating at 2.5 Å to 3.2 Å (Figure 8(a)). A peak 3.7 Å was observed at 45 ns after that started declining and 3.2 Å was recorded at 80 ns, which favors the stability and reliability of the complex. Root-mean-square-fluctuations (RMSF) were calculated in order to understand the fluctuation of individual residue in the docked complex. The results revealed fluctuation peaks for glycine 50, aspartic acid 125, and threonine 275 at 4.8 Å, 4.3 Å, and 4.7 Å, respectively. The high fluctuation in the docked complex residues might be due to the free movement of the residues. Most of the residue in docked protein showed a steady behavior which might indicate the stable interaction with the ligand (Figure 8(b)). B-factor and RMSF are interconvertible and related to each other [35]. The amino acid fluctuations shown by B-factor were similar to RMSF results (Figure 8(c)). The result revealed a very consistent behavior in terms of Rg value between 21.5 and 21.3 Å throughout the MD simulation time (Figure 8(d)).

## 4. Discussion

The phytopathogenic fungi are a serious threat for agriculture sector worldwide [18]. The indiscriminate use of fungicides especially on vegetable and fruit is very harmful for human health [36]. Moreover, it causes environmental pollution and pesticide resistance but these issues are least addressed for fungicides as compared to insecticides [37, 38]. The use of alternate plant fungal disease management methods is need of time. PEOs are volatile molecules produced by plants as secondary metabolites having antifungal potential [39]. The biodegradable nature of PEOs makes them potential candidate for fungicide development [40].

The effectiveness of PEOs, i.e., *Syzygium aromaticum*, *Azadirachta indica*, *Mentha spicata*, *Trachyspermum ammi*, *Moringa oleifera*, *Cuminum cyminum*, *Eucalyptus grandis*, *Allium sativum*, and *Citrus sinensis*, has been tested, against *Fusarium oxysporum*, in the present study. The study was conducted in three trials, i.e., laboratory testing of PEO, field evaluation, and computer-aided fungicide design. MIC and MFC of PEOs were evaluated in laboratory. The MIC of a chemical is its lowest concentration required to inhibit the substantial growth of a pathogen [41]. The lowest MIC and MFC against *F. oxysporum* have been recorded for *Citrus sinensis* PEO. The second effective PEOs were of *E. grandis* and *A. sativum.* Shafique et al. reported good potential of *E. grandis* against different fungi but least effective against other *F. oxysporum* [42]. *Eucalyptus grandis* essential oil was found effective against *F. oxysporum* and *Botrytis cinerea* in banana [43]. Antifungal activity of essential oil from *A. sativum* has been reported from many recent studies [44]. The third runner-up of the treatments was *C. cyminum* (50 *μ*l/ml). Romagnoli et al. and Mohammadpour et al. reported antifungal potential of *C. cyminum* [45, 46]. Three qualified PEOs, *C. sinensis*, *C. cyminum*, and *E. grandis*, from lab experiment were selected to be tested on tomato plants in field conditions. Two trials for field testing were carried out, i.e., foliar application of PEO and seed treatment with PEO. In foliar application trial, the lowest percent disease incidence and percent disease severity were observed for *C. sinensis* followed by *E. grandis*. Percent disease severity was calculated to find out the PEDC of PEO suggested by Jadon et al. [47]. This parameter is used to test the effectiveness of a chemical against pathogenic disease [45]. PEDC of *C. sinensis* was found the highest in our study. The efficacy of PEO was found to be more persistent as seed treatment than as foliar application. It might be possible that when exposed to sunlight the essential oil degrades rapidly [48].

CADD became a popular method for developing new pharmaceutical drugs [49]. Nevertheless, while the pharmacodynamics and techniques used in CADD and pesticide design (CAPD) are similar, this technique is not used in agricultural pesticides [50]. In the subject of pesticide chemistry, research into new target locations and innovative pharmacological compounds is quite limited. A little work has been done in CAPD against phytopathogenic fungi control [51]. Novel, more effective, and least toxic drug molecules and new target sites in the pathogen can be identified more efficiently by using CAPD approaches. This may help to overcome the problem of environment pollution, human health hazards, and pesticide resistance issues in a smart, least expensive, and rapid manner.

The target enzyme of *F. oxysporum* polyketide synthase was selected to be inhibited. It is involved in mediating fusaric acid biosynthesis which is a mycotoxin with low to moderate toxicity to humans and animals but highly phytotoxic [33]. Protein domain is the conserved sequence of protein which controls its function independently [52]. It is better to identify the toxin-producing domain in protein for inhibition by ligand molecules so beta-ketoacyl synthase domain (IPR020841) was selected for inhibition. This domain was found be involved in a number of enzymatic systems, including fatty acid synthetase, which catalyzes the formation of long-chain fatty acids from acetyl-CoA, malonyl-CoA, and NADPH which is involved in the biosynthesis polyketide synthase. Top seven compounds from *C. sinensis* PEO showing minimum binding energy and good hydrogen bonding with target protein were selected for merging their features to construct the pharmacophore. The pharmacophore defined by IUPAC is “a collection of steric and electronic characteristics that is essential to make sure the optimal supramolecular interactions with a particular biological target and to activate (or block) its biological reaction.” It is generally done by retrieving widely used chemical characteristics from 3D structures of a set of known ligands that are reflective of the ligands' crucial interactions with certain macromolecular targets [53]. This can be used as a query for retrieving potential lead identification from structural databases, for designing molecules with specific desired characteristics [54]. This approach has been effectively used in drug designing from novel and human safe chemical compounds [55]. These common features among the top compounds shown by pharmacophore can be used to design a novel fungicide molecule effective against *F. oxysporum.*

High-throughput screening (HTS) has become an integral part of CADD procedure pipeline for identification of hits of effective compounds against target protein [56]. The first step of CADD procedure pipeline is the identification of molecular targets (natural or synthetic) for our protein of interest, and after identification and validation, the selected compound is referred as lead molecule. The molecules found in natural products play a highly significant role in the drug discovery and development process. The chemical compounds that originated from marine organisms are getting attention and becoming popular to be selected as drug molecules [57]. In the current study, we did virtual screening of the pharmacophore, against Comprehensive Marine Natural Products Database (CMNPD). This database contains >38000 marine organism-originated chemical compounds.

Molecular dynamics (MD) simulations were used to check the stability of docked complexes. This analysis of MD simulation is used to evacuate the movements of the highly complexed macromolecular systems [58]. The estimation of structural fluctuations, in terms of RMSD and RMSF, of docked complex is the most crucial feature of this analysis which reflects the stability and flexibility of the complex. The stability interaction profile is reflected in the RMSD value. The average ligand-receptor RMSD in this investigation was 1 nm, showing that the system was stable. The dislocation of a single atom, or a group of atoms, relative to the reference structure is estimated using RMSF, which is averaged across the number of atoms [59].

The compound CMNPD24498 (FW054-1) has been selected as lead molecule for fungicide development against *F. oxysporum*. This compound has been derived from Verrucosispora genus of Micromonosporaceae family [60]. Micromonosporaceae belongs to the gram-positive Actinobacteria. This genus is getting attention in the field of drug development due to antibiotic nature of some species [61]. Some important antibiotic products of *Verrucosispora* sp. are gifhornenolones A and B from *Verrucosispora gifhornensis* [62], thiocoraline A from *Verrucosispora sp.* WMMA107, proximicins A-C from *Verrucosispora fiedleri* MG-37, brevianamide F from *Verrucosispora sp.* MS100047, and butrepyrazinone from *Verrucosispora sp.* K51G [63–65].

## 5. Conclusion

The fungal plant diseases are a very serious threat to fruit and vegetable industry. Complete eradication of disease from plants is required for good market value of fruit and vegetable. Synthetic fungicides are being extensively used for controlling fungal disease. This indiscriminate and extensive use of synthetic chemical compounds causes very serious threat to human health and environment. Alternative and safer pest management methods have been recommended to be tested against phytopathogenic fungi, especially in fruits and vegetables. The use of plant essential oil (PEO) is considered a safe and environment-friendly plant disease control method. In the current study, different plant essential oils have been tested, in the laboratory and field, against *F. oxysporum* in tomato. The plant essential oil of *Citrus sinensis* has been found most effective among all the PEOs tested. But the use of plant essential oil on a large scale for plant disease control is not a cost-effective method. The computer-aided fungicide design (CAFD) technique has been employed to identify safer and effective chemical molecules to be used in fungicide development against *F. oxysporum*. The shared features of the top seven compounds of *C. sinensis* PEO against toxin-producing enzyme, polyketide synthase, of *F. oxysporum* were screened against the CMNPD database. One compound CMNPD24498 (FW054-1) from *Verrucosispora* sp. (bacteria) showed the highest rank of similarity for shared features of selected effective *C. sinensis* PEO compounds. This biological originated compound fulfilled the pesticide likeness criteria and nontoxic in nature. FW054-1 can be used for the development of an effective and safe fungicide against *F. oxysporum*.

## Figures and Tables

**Table 1:** Effect of foliar application of different concentrations of plant essential oils on percent disease severity of *Fusarium oxysporum* in tomato.

No.	Plant essential oil concentrations(*μ*l/ml)	Total leaf area (cm^2^)	Infested leaf area (cm^2^)	PDS^∗∗^	Infested leaf area (cm^2^)	PDS^∗∗^	Reduction	DSS^∗∗∗^	PDI^∗∗∗∗^	PEDC^∗∗∗∗∗^
T1	*Eucalyptus grandis* (80)	23.2	1.5	5.7 ± 0.01^cd^^∗^	0.9	3.7 ± 0.02^c^^∗^	2.6	2-1	40-20	60-80
T2	*Eucalyptus grandis* (60)	22.7	1.3	6.5 ± 0.01^c^	0.9	3.9 ± 0.02^c^	2.0	2-1	40-20	60-80
T3	*Cuminum cyminum* (80)	23.1	2.3	10.0 ± 0.03^b^	2.6	11.3 ± 0.03^b^	-1.3	3-4	20	80
T4	*Cuminum cyminum* (60)	23.3	3.0	12.9 ± 0.01^b^	2.8	12.0 ± 0.02^b^	0.9	4	60	40
T5	*Citrus sinensis* (80)	22.6	0.7	3.1 ± 0.01^d^	0.6	2.7 ± 0.01^d^	0.4	1	20	80
T6	*Citrus sinensis* (60)	22.6	1.0	4.4 ± 0.02^cd^	0.8	3.5 ± 0.02^cd^	-1.8	1	20	80
T7	Ridomil Gold WG	22.8	0.5	2.2 ± 0.02^d^	0.3	1.5 ± 0.03^de^	0.7	1	20	80
T8	Ethanol	23.1	17.0	73.6 ± 0.00^a^	16.0	69.3 ± 0.02^a^	4.3	5	100	0
**LSD**				**1.505**		**1.812**				

^∗^Values sharing the same letters in column are not significantly different. ^∗∗^Percent disease severity. ^∗∗∗^Disease severity scale. ^∗∗∗∗^Percent disease index. ^∗∗∗∗∗∗^Percent efficacy of disease control.

**Table 2:** Effect of seed treatment of different plant essential oils on percent disease severity of *Fusarium oxysporum* in tomato.

No.	Plant essential oils (60 *μ*l/ml)	Total leaf area (cm^2^)	Infected area (cm^2^)	PDS^∗∗^	DSS^∗∗∗^	PDI^∗∗∗∗^	PECD^∗∗∗∗∗^	%incidence
1	*Eucalyptus grandis*	22.0	1.1	3.64 ± 0.1^cd^^∗^	1	40	60	2.6.±0.1^c^
2	*Cuminum cyminum*	21.7	2.7	12.44 ± 0.0^b^	3	60	40	4.5 ± 0.2^b^
3	*Citrus sinensis*	21.6	0.8	2.11 ± 0.3^d^	1	20	80	1.5 ± 0.2^d^
4	Ridomil Gold WG	22.0	0.3	1.36 ± 0.1^d^	1	20	80	1.0 ± 0.2^d^
5	Distilled water	23.0	19.0	82.61 ± 0.1^a^	5	100	0	9.6 ± 0.0^a^
			**LSD**	**1.8725**				**0.4245**

^∗^Values sharing the same letters in column are not significantly different. ^∗∗^Percent disease severity. ^∗∗∗^Disease severity scale. ^∗∗∗∗^Percent disease index. ^∗∗∗∗∗∗^Percent efficacy of disease control.

**Table 3:** Properties of chemical compounds of *Citrus sinensis* essential oil and results of docking with polyketide synthase beta-ketoacyl synthase domain in *Fusarium oxysporum.*

No.	Compounds	PubChem CID	MW (g/mol)	HBD	HBA	nRTB	Logp	BA (kcal/mol)	H-bond
*Monoterpenes*									
1	*β*-Pinene	14896	136.23	1	0	0	3.1	-4.3	0
2	*β*-Myrcene	31253	142.27	1	0	0	4.3	-3.8	0
3	Limonene	22311	136.23	2	0	1	3.4	-5.8	0
4	Sabinene	18818	136.23	1	0	1	3.1	-4.8	0
*Oxygenated monoterpenes*									
5	*α*-Terpineol	17100	154.25	1	1	1	1.8	-4.7	1
6	(Z)-p-Menth-2-en-1-ol	13918681	154.25	1	1	1	2.3	-6.1	1
7	Neral	643779	152.23	0	1	4	3.0	-5.9	1
*Sesquiterpenes*									
8	*δ*-Cadinene	92313	204.35	2	0	1	4.3	-5.7	0
9	*β*-Farnesene	5281517	204.35	1	0	7	6.2	-5.0	0
10	*α*-Cyperone	6452086	218.33	0	1	1	3.8	-5.8	0
11	Caryophyllene oxide	1742210	220.35	0	1	0	3.6	-6.2	0
12	Germacrene B	5281519	204.35	2	0	1	4.7	-6.2	0
*Oxygenated sesquiterpenes*									
13	Elemol	92138	222.37	1	1	3	4.4	-5.3	1
14	Nootkatone	1268142	218.33	0	1	1	3.9	-6.0	2
*Other oxygenated compounds*									
15	Citronellyl acetate	9017	198.3	0	2	7	3.8	-4.8	1
16	Neryl acetate	1549025	196.29	0	2	6	3.5	-4.5	1

MW = molecular weight; HBD = hydrogen bond donor; HBA = hydrogen bond acceptor; nRTB = number of rotatable bonds; BA = binding affinity.

**Table 4:** Properties of marine life-derived compounds and results of docking with polyketide synthase beta-ketoacyl synthase domain in *Fusarium oxysporum.*

No.	CMNPD ID	IUPAC name	MW (g/mol)	Logp	HBA	HBD	nRB	nAR	BA (kcal/mol)	H-bond	Source species
1	91	2-[(1R,2S)-1,2-Dimethyl-3-methylidenecyclopentyl]-5-methylphenol	195.99	3.792	1	0	1	1	-6.9	4	*Laurencia subopposita*
2	538	(1S,2R,3R,5R,6R,7R,8R)-5-[(4E)-6-Hydroxy-6-methylhepta1,4-dien-2-yl]-2,8-dimethyltricyclo[5.3.0.02,6]decan-3-ol	275.01	4.488	2	0	4	0	-4.1	3	*Stoechospermum polypodioides*
3	1118	(3bR,6S,7S,9aR)-7-Hydroxy-6-(hydroxymethyl)-3b,6,9a-trimethyl-4,5,5a,7,9,9b,10,11-octahydronaphtho[2,1-e][2]benzofuran-8-one	303.98	3.25	4	0	1	1	-6.5	2	*Spongia* sp.
4	2024	(1Z)-1-[(3S,3aS,7S,7aS)-3,7-Dimethyl-1,2,3,3a,5,6,7,7a-octahydroinden-4-ylidene]-2-methylpropan-2-ol	198.01	4.752	1	0	1	0	-5.4	1	*Unidentified* sp.
5	2365	(1R,2R,4S)-4-Methyl-1-propan-2-yl-3,4-dihydro-2H-naphthalene-1,2,6-triol	215.98	1.167	3	0	1	1	-5.1	2	*Lemnalia cervicornis*
6	2924	2-[(1R,3E,7E,11E)-4,8,12-Trimethylcyclotetradeca-3,7,11-trien-1-yl]propane-1,2-diol	271.99	4.881	2	0	2	0	-5.8	2	*Sinularia mayi*
7	4785	3-Hydroxy-3,5,5-trimethyl-7-propan-2-yl-2,4-dihydroinden-1-one	211.99	2.328	2	0	1	0	-5.8	1	*Sarcophyton trocheliophorum*
8	5394	[(1R,2aR,3S,4aR,5R,6S,7R,8R,8aR)-3,6-Dibromo-7-hydroxy-2a,4a,5,8-tetramethyl-1,2,3,4,5,6,7,8-octahydrocyclobuta[i]inden-1-yl] acetate	409.82	4.625	3	0	2	0	-5.5	0	*Laurencia tenera*
9	15648	(9Z,11E)-Tricyclo[12.3.1.12,6]nonadeca-1(17),2,4,6(19),9,11,14(18),15-octaene-3,8,17-triol	275.98	2.135	3	0	0	2	-6.7	0	*Cymodocea nodosa*
10	15845	6,7,9a-Trimethyl-1,3,5,5a,6,8,9,10a-octahydrofuro[3,4-b][1] benzoxepin-7-ol	227.98	1.386	3	0	0	0	-5.6	0	*Laurencia luzonensis*
11	18682	(1S,4R,4aS,8aS)-6-(Hydroxymethyl)-4-(2-hydroxypropan-2-yl)-1-methyl-3,4,4a,7,8,8a-hexahydro-2H-naphthalen-1-ol	230	0.841	3	0	2	0	-5.7	3	*Streptomyces* sp.
12	19958	[(1S,3aS,4R,8S,8aR)-8-Ethyl-1,4-dimethyl-3a,5,6,7,8,8a-hexahydro-1H-azulen-4-yl] methanol	198.01	4.216	1	0	2	0	-6.7	1	*Ulva lactuca*
13	20001	(1S,3aR,4S,8aS)-1,4-Dimethyl-7-propan-2-yl-2,3,3a,5,6,8a-hexahydroazulene-1,4-diol	214.01	2.613	2	0	1	0	-5.5	0	*Grateloupia turuturu*
14	20352	(1S,3aS,4S,7S)-1,4-Dimethyl-7-propan-2-yl-3,4,5,6-tetrahydro-2H-azulene-1,3a,7-triol	227.98	0.744	3	0	1	0	-5.8	7	*Sinularia leptoclados*
15	20360	(3S,4E,6S,8Z,11S,16R)-6,16-Dihydroxy-8-(hydroxymethyl)-4,15,15-trimethyltricyclo [9.3.1.13,14] hexadeca-1(14),4,8-trien-2-one	306	0.45	4	0	1	0	-6.3	1	*Cespitularia hypotentaculata*
16	22108	(1S,4S,5S,9R)-8-(Hydroxymethyl)-4-(1-hydroxypropan-2-yl)-1-methylspiro [4.5] dec-7-en-9-ol	228.99	2.351	3	0	3	0	-5.1	5	*Lemnalia cervicornis*
17	**24498**	**2-Hydroxy-3,6-dimethyl-N-(4,5,6-trihydroxy-4-methyloxan-3-yl) benzamide (FW054-1)**	**289.97**	**0.733**	**7**	**0**	**3**	**1**	**-6.2**	**3**	***Verrucosispora* sp.**
18	24574	[(1S,2S,4E,6R,7E,9R)-1,6-Dihydroxy-8-(hydroxymethyl)-4,11,11-trimethyl-2-bicyclo [7.2.0] undeca-4,7-dienyl] acetate	284.98	0.006	5	0	3	0	-5.6	5	*Ascotricha* sp.
19	26841	(1R,2S,5R,8R,9R)-1,4,4,8-Tetramethyl-12-oxatricyclo [6.3.1.02,5] dodecan-9-ol	214.01	3.563	2	0	0	0	-5.2	1	*Rumphella antipathes*
20	26845	(3aS,5S,5aR,6S,8S)-5a,6,8-Trihydroxy-3a-methoxy-1,5,8-trimethyl-4,5,6,7-tetrahydroazuleno[6,5-b] furan-2-one	287.97	-0.833	6	0	1	0	-6.0	0	*Menella kanisa*
21	28987	9-Hydroxy-3-(1-hydroxyethyl)-3H-2-benzoxepin-1-one	207.98	0.862	4	0	1	1	-5.5	4	*Pestalotia heterocornis*
22	30788	(1S,2R,3aR,8aS)-1-(2-hydroxypropan-2-yl)-3a,6-Dimethyl-2,3,4,7,8,8a-hexahydroazulene-1,2-diol	228.99	3.074	3	0	1	0	-5.7	2	*Trichoderma virens*
23	30789	(1S,2R,3aR,4R,8aS)-3a,6-Dimethyl-1-propan-2-yl-2,3,4,7,8,8a-hexahydroazulene-1,2,4-triol	228.99	1.947	3	0	1	0	-6.2	2	*Trichoderma virens*
24	20359	(3S,4E,6R,7S,11R,16R)-6,7,16-Trihydroxy-4,15,15-trimethyl-8-methylidenetricyclo [9.3.1.13,14] hexadeca-1(14),4-dien-2-one	306	1.197	4	0	0	0	-5.4	6	*Cespitularia hypotentaculata*
25	11537	[(1S,8aS)-5,5,8a-Trimethyl-2-methylidene-3,4,4a,6,7,8-hexahydro-1H-naphthalen-1-yl] methanol	195.99	3.067	1	0	1	0	-5.6	0	*Cadlina luteomarginata*
26	11370	(1R,3aR,4R,8aR)-1-Methoxy-1,4-dimethyl-7-propan-2-yl-2,3,3a,5,6,8a-hexahydroazulen-4-ol	226.01	3.132	2	0	2	0	-5.8	0	*Sarcophyton buitendijki*
27	5390	2-[(1R,3R,4S)-4-Chloro-1,3-dimethylcyclohexyl]-5-methylphenol	230.96	3.789	1	0	1	1	-6.7	0	*Laurencia dendroidea*

MW = molecular weight; HBD = hydrogen bond donor; HBA = hydrogen bond acceptor; nRTB = number of rotatable bonds; BA = binding affinity; nAR = number of aromatic rings.

## Data Availability

The authors declare that all the data supporting the findings of this study are included in the article.
