# Supplementary material for: Identification of Novel and Safe Fungicidal Molecules against Fusarium oxysporum from Plant Essential Oils: In Vitro and Computational Approaches
Source: Biomed Res Int. 2022 Jul 26;2022:5347224. doi: 10.1155/2022/5347224 (PMC9345698; doi:10.1155/2022/5347224)
Supplement: Supplementary Materials — The supplementary file S1 is available online along with the manuscript. Other supplementary material will be provided by the corresponding author on request. [file 5347224.f1.docx]

| **Treatment** | **Plant Names** | **Technical Names** | **Concentration**  **(µl/ml)** |
| --- | --- | --- | --- |
| T1 | Clove | *Syzygium aromaticum* | 25 |
| T2 | Clove | *Syzygium aromaticum* | 50 |
| T3 | Cumin | *Cuminum cyminum* | 25 |
| T4 | Cumin | *Cuminum cyminum* | 50 |
| T5 | Moringa | *Moringa olifera* | 25 |
| T6 | Moringa | *Moringa olifera* | 50 |
| T7 | Carom seeds | *Trachyspermum ammi* | 25 |
| T8 | Carom seeds | *Trachyspermum ammi* | 50 |
| T9 | Mint | *Mentha spicata* | 25 |
| T10 | Mint | *Mentha spicata* | 50 |
| T11 | Eucalyptus | *Eucalyptus grandis* | 25 |
| T12 | Eucalyptus | *Eucalyptus grandis* | 50 |
| T13 | Garlic | *Allium sativum* | 25 |
| T14 | Garlic | *Allium sativum* | 50 |
| T15 | Sweet orange | *Citrus sinensis* | 25 |
| T16 | Sweet orange | *Citrus sinensis* | 50 |
| T17 | Azadirachta | *Azadirachta indica* | 25 |
| T18 | Azadirachta | *Azadirachta indica* | 50 |
| T19 | Control | Ethanol |  |

**Supplementary Table 1: Treatment of plant essential oils with different concentrations to test MIC against *Fusarium oxysporum***

**Supplementary Table 2: Treatment of Plant essential oils with different concentrations for foliar application against *Fusarium oxysporum***

| **Treatment** | **Plant Names** | **Technical Names** | **Concentration**  **(µl/ml)** |
| --- | --- | --- | --- |
| T1 | Cumin | *Cuminum cyminum* | 60 |
| T2 | Cumin | *Cuminum cyminum* | 80 |
| T3 | Caraway | *Trachyspermum ammi* | 60 |
| T4 | Caraway | *Trachyspermum ammi* | 80 |
| T5 | Sweet orange | *Citrus sinensis* | 60 |
| T6 | Sweet orange | *Citrus sinensis* | 80 |
| T7 | -ve Control | Ethanol |  |
| T8 | +ve Control | Ridomil Gold (WG) |  |

**Supplementary Table 3:** Chemical compound found in different organs of citrus

| Ser. | Compound Name | Class | Organ | Bioactivity | References |
| --- | --- | --- | --- | --- | --- |
| 1 | α -pinene | monoterpene | flower, peel, leaf | antimicrobial | Hosni et al. (2010), Lota et al. (2002), Jabalpurwala et al. (2009), Sartorelli et al. (2007) |
| 2 | α-thujene | monoterpene | flower, peel, leaf |  | Jabalpurwala et al. (2009), Lota et al. (2002) |
| 3 | camphene | monoterpene | flower, leaf | lipid lowering | Lota et al. (2002), Bourgou et al. (2012), Vallianou et al. (2011), Sawamura et al. (1991) |
| 4 | β-pinene | monoterpene | flower, peel, leaf | antifungal | Jabalpurwala et al. (2009), Lota et al. (2002), Hammer et al. (2003) |
| 5 | sabinene | monoterpene | flower, peel, leaf | antifungal | Hosni et al. (2010), Lota et al. (2002), Jabalpurwala et al. (2009), Espinosa-garcia and Langeneim (1991) |
| 6 | α-phellandrene | monoterpene | peel, leaf | insecticidal activity | Hosni et al. (2010), Park et al. (2003) |
| 7 | δ-3 Carene | monoterpene | flower, peel, leaf | anti-inflammatory | Hosni et al. (2010), Lota et al. (2002), Jabalpurwala et al. (2009), Ocete et al. (1989) |
| 8 | α-myrcene | monoterpene | flower, peel, leaf |  | Hosni et al. (2010), Lota et al. (2002) |
| 9 | β-myrcene | monoterpene | flower, leaf | embryofoetotoxicity antifungal | Jabalpurwala et al. (2009), Lota et al. (2002), Araujo et al. (1996), Tao et al. (2013) |
| 10 | α-terpinene | monoterpene | flower, peel, leaf |  | Jabalpurwala et al. (2009), Lota et al. (2002) |
| 11 | γ-terpinene | monoterpene | flower, peel, leaf | lipid lowering, antimicrobial | Jabalpurwala et al. (2009), Lota et al. (2002), Sartorelli et al. (2007), Takahashi et al. (2003) |
| 12 | limonene | monoterpene | flower, peel, leaf | anti-inflammatory, antioxidant, antidiabetic, anticancer, lipid lowering | Shaw (1979), Hosni et al. (2010), Lota et al. (2002), Jabalpurwala et al. (2009), Jing et al. (2013) |
| 13 | 1,8-cineole | monoterpene oxide | leaf | antivascular, anti-inflammatory | Lota et al. (2002), Santos and Rao (2000), Lahlou et al. (2002) |
| 14 | (Z)-ocimene | monoterpene | flower, peel, leaf |  | Hosni et al. (2010), Lota et al. (2002), Jabalpurwala et al. (2009) |
| 15 | (E)-ocimene | monoterpene | flower, peel, leaf |  | Hosni et al. (2010), Lota et al. (2002), Jabalpurwala et al. (2009) |
| 16 | Trans-sabinene hydrtae A |  | peel, leaf |  | Hosni et al. (2010),), Lota et al. (2002), Vekiari et al. (2002) |
| 17 | Cis-sabinene  Hydrate A |  | peel |  | Hosni et al. (2010) |
| 18 | p-cymene | monoterpene | flower, peel, leaf | antimicrobial, lipolytic effect | Lota et al. (2002), Vekiari et al. (2002), Sartorelli et al. (2007), Lota et al. (2001), Choi (2006) |
| 19 | α-terpinolene | monoterpene | flower, peel, leaf | radical-scavenging activity | Hosni et al. (2010), Lota et al. (2002), Jabalpurwala et al. (2009), Choi et al. (2000) |
| 20 | linalool | monoterpene alcohol | flower, peel, leaf | antidiabetic | Hosni et al. (2010), Lota et al. (2002), Jabalpurwala et al. (2009), Deepa and Anuradha (2011) |
| 21 | Trans-pinocarveol |  | peel |  | Hosni et al. (2010) |
| 22 | Neo-alloocimene | monoterpene | flower |  | Jabalpurwala et al. (2009) |
| 23 | Allo-ocimene | monoterpene | flower |  | Jabalpurwala et al. (2009) |
| 24 | (Z)-epoxy-ocimene |  | flower |  | Jabalpurwala et al. (2009) |
| 25 | Terpinen-4-ol | monoterpene oxide | leaf | antihypertension, anti-inflammatory | Lota et al. (2002), Lahlou et al. (2003), Hart et al. (2000) |
| 26 | β-cyclocitral | sesquiterpenoid | peel | antimicrobial | Hosni et al. (2010), Proszenyak et al. (2007) |
| 27 | Cis-linalool oxide | monoterpene oxide | leaf |  | Lota et al. (2002) |
| 28 | α – p- dimethylstyrene |  | flower |  | Jabalpurwala et al. (2009) |
| 29 | Sabinene hydrate | monoterpene | flower |  | Jabalpurwala et al. (2009) |
| 30 | Trans-para-menth-2-ene-1-ol | monoterpene alcohol | peel |  | Hosni et al. (2010) |
| 31 | Carvacryl methyl oxide | monoterpene phenol derivative | peel |  | Hosni et al. (2010) |
| 32 | (Z)-limonene oxide | monoterpene oxide | flower |  | Jabalpurwala et al. (2009) |
| 33 | δ-elemene | sesquiterpene hydrocarbon | flower | anticancer | Jabalpurwala et al. (2009) |
| 34 | β -elemene | sesquiterpene hydrocarbon | flower, peel, leaf | anti-glioblastome proliferation | Lota et al. (2002), Zhu et al. (2011), Jabalpurwala et al. (2009) |
| 35 | α -copaene | sesquiterpene hydrocarbon | flower, peel | Attractant for male fruit flies | Lota et al. (2002), Nishida et al. (2000) Jabalpurwala et al. (2009) |
| 36 | β -copaene | sesquiterpene hydrocarbon | peel |  | Hosni et al. (2010) |
| 37 | (E)- (E)-2,4-decadienel |  | peel |  | Hosni et al. (2010) |
| 38 | α -bergamotene | sesquiterpene hydrocarbon | flower |  | Jabalpurwala et al. (2009) |
| 39 | trans carveol | monoterpenoid alcohol | peel |  | Hosni et al. (2010) |
| 40 | (E) -caryophyllene | sesquiterpene hydrocarbon | peel, leaf | antimicrobial | Lota et al. (2002), Juliani jr et al. (2002) |
| 41 | β -caryophyllene | sesquiterpene hydrocarbon | flower | anti-inflammatory, antibiotic, anticancer, antioxidant | Legault and Pichette (2007) |
| 42 | trans – α -bergamotene | sesquiterpene hydrocarbon | peel |  | Lota et al. (2002) |
| 43 | β -ionone | isoprenoid | peel | antibiotic, anticancer, | Hosni et al. (2010), Duncan et al. (2004), Radulovic et al. (2006) |
| 44 | β -farnesene | sesquiterpene | flower |  | Jabalpurwala et al. (2009) |
| 45 | (E)- β- farnesene | sesquiterpene | peel | Kairomone for the lady bird | Lota et al. (2002), Francis et al. (2004) |
| 46 | (E, E)- α- farnesene | sesquiterpene | peel | attractant for lepidopteran | Lota et al. (2002), Pechous and Whitaker (2004) |
| 47 | farnesol | sesquiterpene | flower | anticancer, lipid-regulation | Jabalpurwala et al. (2009), Burke et al. (1997), Meigs and Simoni (1997) |
| 48 | α -humulene | sesquiterpene | peel | anticancer | Lota et al. (2002), Hosni et al. (2010), Legault and Pichette (2007) |
| 49 | β -bisabolene | sesquiterpene | flower, peel |  | Lota et al. (2002), Jabalpurwala et al. (2009) |
| 50 | geranial | sesquiterpene aldehyde | flower, leaf | antifungal | Lota et al. (2002), Jabalpurwala et al. (2009), Wuryatmo et al. (2003) |
| 51 | geraniol | terpene alcohol | flower, leaf | anti-inflammatory, antibiotic, anticancer, antioxidant | Lota et al. (2002), Jabalpurwala et al. (2009), Chen and Viljoen (2010) |
| 52 | α -citronellol | monoterpene alcohol | flower, leaf |  | Lota et al. (2002), Jabalpurwala et al. (2009) |
| 53 | β – citronellol | monoterpene alcohol | flower, leaf |  | Lota et al. (2002), Jabalpurwala et al. (2009) |
| 54 | curcumene | sesquiterpene hydrocarbon | flower | anti-inflammatory | Jabalpurwala et al. (2009), Mujumdar et al. (2004) |
| 55 | neral | monoterpene alcohol | peel, leaf | antifungal | Lota et al. (2002), Wuryatmo et al. (2003) |
| 56 | nerol | monoterpene aldehyde | flower, leaf | antimicrobial | Lota et al. (2002), Jabalpurwala et al. (2009), kotan et al. (2007) |
| 57 | calamenene | sesquiterpene | flower | anticancer | Jabalpurwala et al. (2009), Dai et al. (2012) |
| 58 | (Z)- jasmone |  | flower | insecticidal activity | Jabalpurwala et al. (2009), Birkett et al. (2000) |
| 59 | nerolidol | sesquiterpene | flower | insecticidal activity | Lota et al. (2002), Arruda et al. (2005), Jabalpurwala et al. (2009) |
| 60 | thymol | monoterpene phenol | flower | antimicrobial | Jabalpurwala et al. (2009), Betancur-Galvis et al. (2011) |
| 61 | n- phenyl formamide |  |  |  |  |
| 62 | aromadendrene | sesquiterpene | peel | antifungal | Hosni et al. (2010), Hammer et al. (2003) |
| 63 | α -terpineol | monoterpene alcohol | peel, leaf | antifungal | Lota et al. (2002), Hammer et al. (2003) |
| 64 | α -cyperone | sesquiterpene | peel |  | Hosni et al. (2010) |
| 65 | Geranyl α- terpinene |  | peel |  | Hosni et al. (2010) |
| 66 | δ -cadinene | sesquiterpene | peel |  | Hosni et al. (2010) |
| 67 | germacrene-B | sesquiterpene hydrocarbon | peel |  | Hosni et al. (2010) |
| 68 | germacrene-D | sesquiterpene hydrocarbon | peel | insecticidal activity | Hosni et al. (2010), Lota et al. (2002), Rϕstelien et al. (2000) |
| 69 | α -sinensal | sesquiterpene aldehyde | peel, leaf |  | Hosni et al. (2010), Lota et al. (2002) |
| 70 | β -sinensal | sesquiterpene aldehyde | peel, leaf |  | Hosni et al. (2010), Lota et al. (2002) |
| 71 | γ -eudesmol | sesquiterpenoid | peel |  | Hosni et al. (2010) |
| 72 | τ -cadinol | sesquiterpene | peel |  | Hosni et al. (2010) |
| 73 | bicyclogermacrene | sesquiterpene hydrocarbon | peel |  | Hosni et al. (2010) |
| 74 | caryophyllene oxide | sesquiterpene oxide | peel, leaf | antifungal, anti-inflammatory | Lota et al. (2002), Yang et al. (2000), Chavan et al. (2010) |
| 75 | α -calacorene | sesquiterpene | peel |  | Hosni et al. (2010) |

**Supplementary Table 4:** Citrus compounds qualified pesticide likeness and non-hazardous parameters

| **Sr. No** | **Compounds** | **CID** | **Molecular Formula** | **Mol.wt (g/mol)** | **HBD** | **HBA** | **nRtbond** | **Logp** |
| --- | --- | --- | --- | --- | --- | --- | --- | --- |
| **Monoterpenes** | | | | | | | | |
| 1 | Pinene<Alpha-> | 15837102 | C10H16 | 136.23 | 0 | 0 | 0 | 2.6 |
| 2 | Pinene<Beta-> | 14896 | C10H16 | 136.23 | 0 | 0 | 0 | 3.1 |
| 3 | Myrcene<Beta-> | 31253 | C10H16 | 142.27 | 0 | 0 | 0 | 4.3 |
| 4 | Carene<Delta-3-> | 26049 | C10H16 | 136.23 | 0 | 0 | 0 | 2.8 |
| 5 | Limonene | 22311 | C10H16 | 136.23 | 0 | 0 | 1 | 3.4 |
| 6 | Terpinene<Gamma-> | 7461 | C10H16 | 136.23 | 0 | 0 | 1 | 2.8 |
| **Oxygenated Monoterpenes** | | | | | | | | |
| 7 | Linalool | 6549 | C10H18O | 154.25 | 1 | 1 | 4 | 2.7 |
| 8 | Citronellal | 7794 | C10H18O | 154.25 | 0 | 1 | 5 | 5.0 |
| 9 | Terpin-4-ol | 11230 | C10H18O | 154.25 | 1 | 1 | 1 | 2.2 |
| 10 | Terpineol<Alpha-> | 17100 | C10H18O | 154.25 | 1 | 1 | 1 | 1.8 |
| 11 | Nerol | 643820 | C26H30O6 | 154.25 | 1 | 1 | 4 | 2.9 |
| 12 | Neral | 643779 | C26H30O6 | 152.23 | 0 | 1 | 4 | 3.0 |
| 13 | Geraniol | 637566 | C26H30O6 | 154.25 | 1 | 1 | 4 | 2.9 |
| 14 | Geranial | 91750110 | C26H30O6 | 438.25 | 0 | 6 | 7 | 5.9 |
| **Sesquiterpenes** | | | | | | | | |
| 15 | Elemene<Beta-> | 6918391 | C15H24 | 204.35 | 0 | 0 | 3 | 6.1 |
| 16 | Caryophellene<E-> | 5281515 | C15H24 | 220.35 | 0 | 0 | 0 | 4.4 |
| 17 | δ -cadinene | 348293214 | C15H24 | 204.35 | 0 | 0 | 1 | 4.3 |
| 18 | Bergamotene<Alpha-Trans-> | 6429302 | C15H24 | 204.35 | 0 | 0 | 3 | 4.8 |
| 19 | (E)- β- farnesene | 671258 | C15H24 | 204.35 | 0 | 0 | 0 | 3.5 |
| 20 | alpha-Cyperone | 6452086 | C15H22O | 218.33 | 0 | 1 | 1 | 3.8 |
| 21 | Humulene<Alpha-> | 5281520 | C15H24 | 204.35 | 0 | 0 | 0 | 4.5 |
| 22 | Farnesene<(E)-Beta-> | 5281517 | C15H24 | 204.35 | 0 | 0 | 7 | 6.2 |
| 23 | Germacrene B | 5281519 | C15H24 | 204.35 | 0 | 0 | 1 | 4.7 |
| 24 | Valencene | 9855795 | C15H24 | 204.35 | 0 | 0 | 0 | 4.1 |
| 25 | Bisabolene<(Z)-Alpha-> | 5352653 | C15H24 | 204.35 | 0 | 0 | 4 | 5.2 |
| 26 | Bisabolene (Beta-) | 10104370 | C15H24 | 204.35 | 0 | 0 | 3 | 5.2 |
| **Oxygenated Sesquiterpenes** | | | | | | | | |
| 27 | Elemol | 92138 | C15H26O | 222.37 | 1 | 1 | 3 | 4.4 |
| 28 | Nerolidol<E-> | 5284507 | C15H26O | 222.37 | 1 | 1 | 7 | 4.6 |
| 29 | Bisabolol<Alpha-> | 1549992 | C15H26O | 222.37 | 1 | 1 | 4 | 3.8 |
| 30 | Nootkatone | 1268142 | C15H22O | 218.33 | 0 | 1 | 1 | 3.9 |
|  | **Other oxygenated compounds** | | | | | | | |
| 31 | Nonanal<N-> | 31289 | C9H18O | 142.24 | 0 | 1 | 7 | 3.3 |
| 32 | Citronellyl Acetate | 9017 | C9H18O | 198.3 | 0 | 2 | 7 | 3.8 |
| 33 | Neryl Acetate | 1549025 | C12H20O2 | 196.29 | 0 | 2 | 6 | 3.5 |
